# Clinical impact of early microchimerism dynamics after transplantation with enhanced dual-conditioning regimen in hematological diseases

**DOI:** 10.3389/fmed.2025.1726471

**Published:** 2025-12-04

**Authors:** Yan Gu, Shengnan Du, Yilian Yang, Jiahua Ding

**Affiliations:** 1 Department of Geriatrics, The Second Hospital of Nanjing, Nanjing University of Chinese Medicine, Nanjing, China; 2 Department of Hematology, Nanjing Lishui People’s Hospital, Nanjing, China; 3 Department of Hematology, Nanjing Pukou People’s Hospital, Nanjing, China; 4 Department of Hematology, Zhongda Hospital, Southeast University, Nanjing, China; 5 Department of Hematology, Nanjing BenQ Medical Center, Nanjing, China

**Keywords:** microchimerism, enhanced dual-conditioning regimen, engraftment, survival, allo-HSCT

## Abstract

**Background:**

Microchimerism dynamics following allogeneic hematopoietic stem cell transplantation (allo-HSCT) may predict engraftment and clinical outcomes. This study aimed to quantify microchimerism changes and assess their clinical significance.

**Methods:**

In this retrospective study, eighteen patients undergoing allo-HSCT received either an enhanced dual-conditioning (EDCT) regimen (fludarabine/busulfan/cytarabine plus cyclophosphamide 200 mg/kg) or a modified EDCT regimen. Microchimerism levels were serially monitored from day +1 post-transplantation.

**Results:**

Complete donor chimerism (CDC) was achieved in 16/18 patients (88.9%) at a median time of 14 days (range, 9–24). The median neutrophil and platelet engraftment times were 15 days (range, 11–28) and 25 days (range, 10–80 in 16 patients who had platelet engraftment), respectively. Among them, eight patients retained constant CDC, 3 developed one increasing mixed chimerism (IMC), while 5 had multiple IMCs. Patients with constant CDC demonstrated faster platelet engraftment (median, 19.5 vs. 40 days, *P* = 0.066) and superior overall survival (OS, median, not reached vs. 5.0 months, 95% CI 2–10 months, *P* = 0.015). Notably, microchimerism trends differed between peripheral blood stem cell transplantation (PBSCT) and cord blood transplantation (CBT) recipients. The PBSCT group exhibited shorter neutrophil (median: 14.5 vs. 17.5 days, *P* = 0.165) and platelet (median: 15 days vs. 40 days, *P* = 0.009) engraftment times compared to the CBT group. However, the final CDC rates and OS times did not differ significantly between the two groups.

**Conclusion:**

Early microchimerism dynamics correlate with engraftment efficiency and survival outcomes in allo-HSCT patients, suggesting its clinical utility for timely intervention and personalized treatment adjustment. The promising long-term outcomes support the applicability of this regimen and monitoring approach across transplantation modalities.

## Introduction

Allogeneic hematopoietic stem cell transplantation (allo-HSCT) remains a cornerstone therapy for hematological malignancies and refractory non-malignant blood disorders, offering curative potential for patients with otherwise limited treatment options (1, 2). However, its success is frequently hindered by formidable challenges, including graft-versus-host disease (GVHD), disease relapse, and non-relapse death (3–8). To overcome these challenges, we developed an enhanced dual-conditioning (EDCT) regimen based on the BuCy200 protocol and prior research (9, 10), which combines modified cyclophosphamide (200 mg/kg, CY200) with a fludarabine/busulfan/cytarabine (FBA) backbone (11). Clinical validation has demonstrated that this approach significantly improves engraftment and survival.

Chimerism monitoring following allo-HSCT has become a pivotal clinical tool for assessing engraftment dynamics and predicting long-term clinical trajectories (12). Accurate chimerism evaluation enables timely immunomodulatory interventions to mitigate transplantation-related complications (13). Over recent decades, conventional methods have been progressively replaced by advanced techniques with superior detection limits, such as single nucleotide polymorphisms (SNP) analysis, insertion-deletions (Indel) profiling, and next generation sequencing (14–16). Notably, insertion-deletion quantitative polymerase chain reaction (Indel-qPCR), also known as microchimerism analysis, employs polymerase chain reaction (PCR) amplification of Indel polymorphisms (17–19). This method exhibits exceptional sensitivity and specificity, with a detection limit of 0.001% and the capacity to reliably detect minor cell populations below 1%, underscoring its considerable clinical utility (20).

The correlation between microchimerism and relapse remains incompletely understood due to substantial heterogeneity among published studies (20, 21). Although day +28 is universally recognized as the engraftment milestone, the clinical significance of chimerism assessment prior to this timepoint remains uncertain. Furthermore, longitudinal chimerism monitoring using the EDCT regimen after allo-HSCT has not been previously investigated. To bridge these knowledge gaps, we retrospectively analyzed 18 patients to evaluate the clinical utility of early Indel-qPCR-based microchimerism analysis for assessing engraftment and prognosis following the EDCT regimen. Our results characterize post-transplant microchimerism dynamics and demonstrate its association with hematological recovery and survival outcomes.

## Materials and methods

### Patient cohort

This retrospective cohort study included 18 consecutive patients who underwent allo-HSCT at our center between May 2018 and December 2019. The cohort comprised 8 patients receiving peripheral blood stem cell transplantation (PBSCT) and 10 undergoing cord blood transplantation (CBT), with followed-up extending until June 2024. Of the participants, 9 (50%) were diagnosed with malignancies, including 5 cases of acute myeloid leukemia (AML), 2 of acute lymphocytic leukemia (ALL), and 2 of myelodysplastic syndrome (MDS). The remaining 9 patients (50%) had non-malignant disorders: 6 with severe aplastic anemia (SAA), 2 with β thalassemia, and 1 with Wiskot-Aldrich syndrome (WAS). All patients were refractory to multiple lines of therapy or experienced disease recurrence. Detailed demographic characteristics, graft profiles, and conditioning regimens are summarized in Table 1.

**TABLE 1:** Clinical characteristic of the patients.

Patients’ characteristics	n = 18
Median age (range), years	18 (0.8–60)
PBSCT	46 (39–60)
CBT	2.9 (0.8–25)
Median weight (range), kg	42 (8–80)
PBSCT	66 (55–80)
CBT	14.5 (8–56)
**Sex (n, %)**	
Male	11 (61.1)
Female	7 (38.9)
**Stem cell source (n, %)**	
Peripheral blood	8 (44.4)
Cord blood	10 (55.6)
**Type of diseases (n, %)**	
Non-malignancies	9 (50)
Malignancies	9 (50)
**Conditioning regimen**	
Non-malignancies	F/FB plus CY200
Malignancies	FBA plus CY200
**GVHD prophylaxis**	
PBSCT	CSA + MMF + MTX ± MP
CBT	CSA + MMF

PBSCT, peripheral blood stem cell transplantation; CBT, cord blood transplantation; FBA plus CY200, fludarabine + busulfan + cytarabine + cyclophosphamide; GVHD, graft versus host disease; MTX, methotrexate; CSA, cyclosporin A; MMF mycophenolate mofetil; MP, methylprednisolone.

### Transplantation procedure

All patients underwent either EDCT or modified EDCT regimens. For non-malignancies, the conditioning regimen consisted of F/FB combined with CY200, whereas malignancies were treated with FBA plus CY200 (9, 11). In mitigate GVHD, CBT recipients received cyclosporin A (CsA, days −1 to +180) and mycophenolate mofetil (MMF, days −1 to +28, extended in cases of acute GVHD), wihle PBSCT patients were supplemented with methotrexate (MTX) and methylprednisolone as clinically indicated.

### Quantitative microchimerism detection

Early post-transplant microchimerism was quantitated using Indel-qPCR. Pre-transplant peripheral blood (PB) samples from both recipients and donors were systematically collected to identify informative genetic markers, ensuring specificity for subsequent analysis. Post-transplant PB samples were periodically obtained from recipients from day +1 until at least day +30, with mononuclear cells (MNCs) isolated via Ficoll density gradient centrifugation and genomic DNA subsequently extracted. The Indel-qPCR assay employed a minimum of two recipient-specific markers, which were previously validated as absent in the donor genome, to ensure robust discrimination. Chimerism levels of recipients were quantified using the ΔΔCt method (22), where ΔΔCt = (Ct_informative marker (post–HSCT)_ − Ct_Actin (post–HSCT)_) − (Ct_informative marker (pre–HSCT)_ − Ct_Actin (pre–HSCT)_), with recipient chimerism rate (%) derived from the equation 2^−ΔΔCt^ × 100%. Final chimerism rates were determined by averaging results across all informative markers.

### Study endpoints and definitions

The primary endpoints of this study were engraftment time and overall survival (OS). Engraftment time was quantified as the number of days post-transplantation required to achieve either neutrophil recovery (defined as an absolute neutrophil count > 0.5 × 10^9^/L sustained for 3 consecutive days) or platelet recovery (defined as an untransfused platelet count > 20 × 10^9^/L maintained for 7 consecutive days). OS was calculated from the day of transplantation (day 0) until death from any cause or the last confirmed follow-up. Acute GVHD was diagnosed and graded according to established consensus criteria (23).

To further characterize post-transplant dynamics, we defined complete donor chimerism (CDC) as the presence of ≤0.1% recipient-derived cells (indicative of microchimerism). Increasing mixed chimerism (IMC) was identified as a ≥0.1% rise in recipient cell proportion relative to the prior measurement. Single IMC events were demarcated by transient increases in mixed chimerism without subsequent progression, whereas multiple IMCs required at least two discrete episodes of rising recipient cell fractions.

### Statistical analysis

Patient characteristics were presented as frequencies with percentages for categorical variables and as medians with interquartile ranges for non-normally distributed continuous variables. Continuous variables were compared using the Mann-Whitney U test, while categorical variables were analyzed with the χ^2^ test. Survival outcomes were evaluated using Kaplan–Meier curves with log-rank tests for comparison. All statistical analyses were performed using GraphPad Prism v9.0 (GraphPad Software, San Diego, CA) and SPSS v26.0 (IBM Corporation, Armonk, NY, USA). A two-sided *P*-value < 0.05 was considered statistically significant, with all confidence intervals calculated at the 95% level.

### Ethics approval

The Institutional Review Boards (IRB) of Zhongda Hospital of Southeast University approved this study, waiving the requirement for written informed consent in accordance with IRB regulations.

## Results

### Post-HSCT microchimerism dynamics

Peripheral blood samples from in 18 patients who underwent allo-HSCT were retrospectively analyzed for donor microchimerism dynamics. CDC was achieved in 16 patients (88.9%), with a median time to CDC of 14 days (range: 9–24 days). Serial measurements revealed a progressive increase in median donor chimerism: 88.9% (range: 18.8%–97.0%) at day +7, 96.30% (range: 30.7%–99.9%) at day +10, 99.0% (range: 79.6%–99.9%) at day +14, reaching 100% (range: 94.5%–100%) by day +21, and stabilizing at 99.9% (range: 93.4%–100%) by day +28. Among CDC-achieving patients, 8 maintained sustained CDC, whereas 3 and 5 patients exhibited single or multiple events of IMC, respectively. Notably, 2 non-CDC patients (a 3-years-old with β-thalassemia major receiving CBT and a 39-years-old with ALL receiving PBSCT) succumbed within 2 months post-HSCT (Table 2).

**TABLE 2:** Microchimerism dynamics and clinical outcome of patients.

Case (no.)	Days to CDC	Microchimerism Dynamics	Days to neutrophil engraftment	Days to platelet engraftment	aGVHD	OS (months)
1	10	Multiple IMCs	11	13		2
2	17	One IMC	15	21	IV intestinal	7
3	11	Constant CDC	11	10		6
4	16	Constant CDC	16	19		7
5	14	Multiple IMCs	13	80		6
6	23	Constant CDC	19	28	IV intestinal	72+
7	21	One IMC	28	53	IV intestinal	4
8	–	No CDC	15	–		2
9	17	One IMC	20	58		74+
10	24	Constant CDC	20	40		74+
11	11	Constant CDC	16	20		74+
12	9	Multiple IMCs	14	22		76+
13	12	Constant CDC	20	12		75+
14	13	Constant CDC	13	15	II liver	70+
15	21	Constant CDC	25	42		78+
16	–	No CDC	15	50		2
17	13	Multiple IMCs	13	12		2
18	14	Multiple IMCs	14	–		7

CDC, complete donor chimerism; GVHD, graft versus host disease; OS, overall survival; IMC, increased mixed chimerism.

### Microchimerism and hematopoietic recovery

All patients achieved neutrophil engraftment within a median of 15 days (range: 11–28 days), and 16 of 18 patients (88.9%) exhibited platelet recovery at a median of 25 days (range: 10–80 days) post-HSCT. Two patients failed to achieve platelet engraftment: one with persistent IMC after initial CDC, and another never attained CDC. Notably, patients maintaining stable CDC (*n* = 8) exhibited comparable neutrophil engraftment times (median: 14.5 days; range: 11–25 days) to other patients (median: 17.5 days; range: 11–28 days; *P* > 0.05), but demonstrated significantly faster platelet recovery (median: 19.5 days [range, 10–42 days] vs. 50 days [range, 13–80 days]; *P* = 0.066), with this difference approaching statistical significance (Table 2).

### Microchimerism predicts post-HSCT survival

**FIGURE 1:**
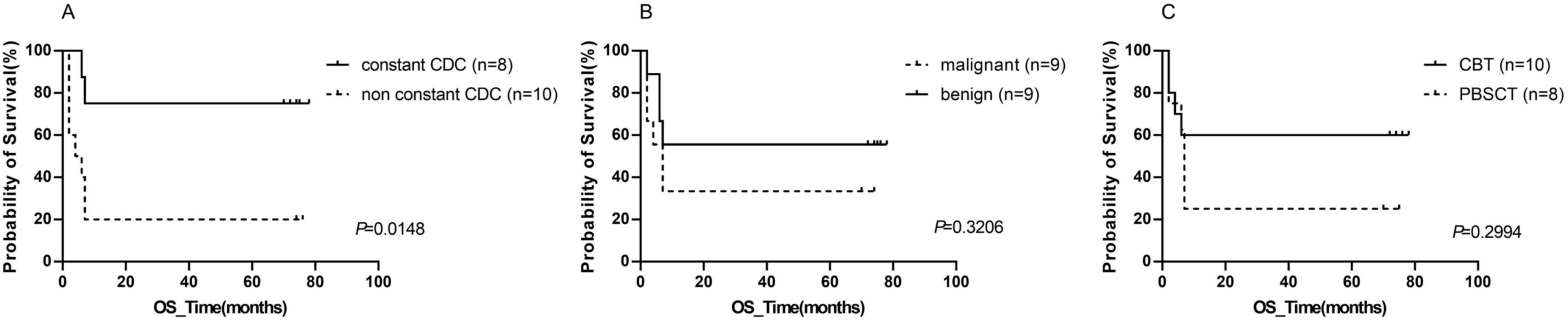
K-M curves according to different groups for overall survival. **(A)** Patients with constant CDC vs. patients without constant CDC; **(B)** patients with malignancies vs. patients with non-malignancies; **(C)** patients received PBSCT vs. patients received CBT.

At the end of the observation period, 10 of 18 patients (55.6%) had died. Primary causes of death comprised relapse, platelet engraftment failure, and transplantation-related non-relapse mortality. Notably, 6 of 8 patients with sustained CDC survived, whereas the remaining 2 succumbed to cytomegalovirus (CMV) infection and GVHD. The 3-years and 5-years OS rates were significant higher in the constant CDC group compared with the non-constant CDC group (both 75% vs. 20%, *P* = 0.013), as well as the median OS time [not reached vs. 5.0 months (95% CI 2.0–10.0 months), *P* = 0.015, Figure 1A]. Conversely, OS did not differ significantly between patients with/without malignancies (*P* = 0.321, Figure 1B) or between CBT/PBSCT recipients (*P* = 0.299, Figure 1C). Both patients failing to achieve CDC died within 2 months from platelet-specific engraftment failure.

### Dynamic microchimerism patterns in PBSCT and CBT recipients

**FIGURE 2:**
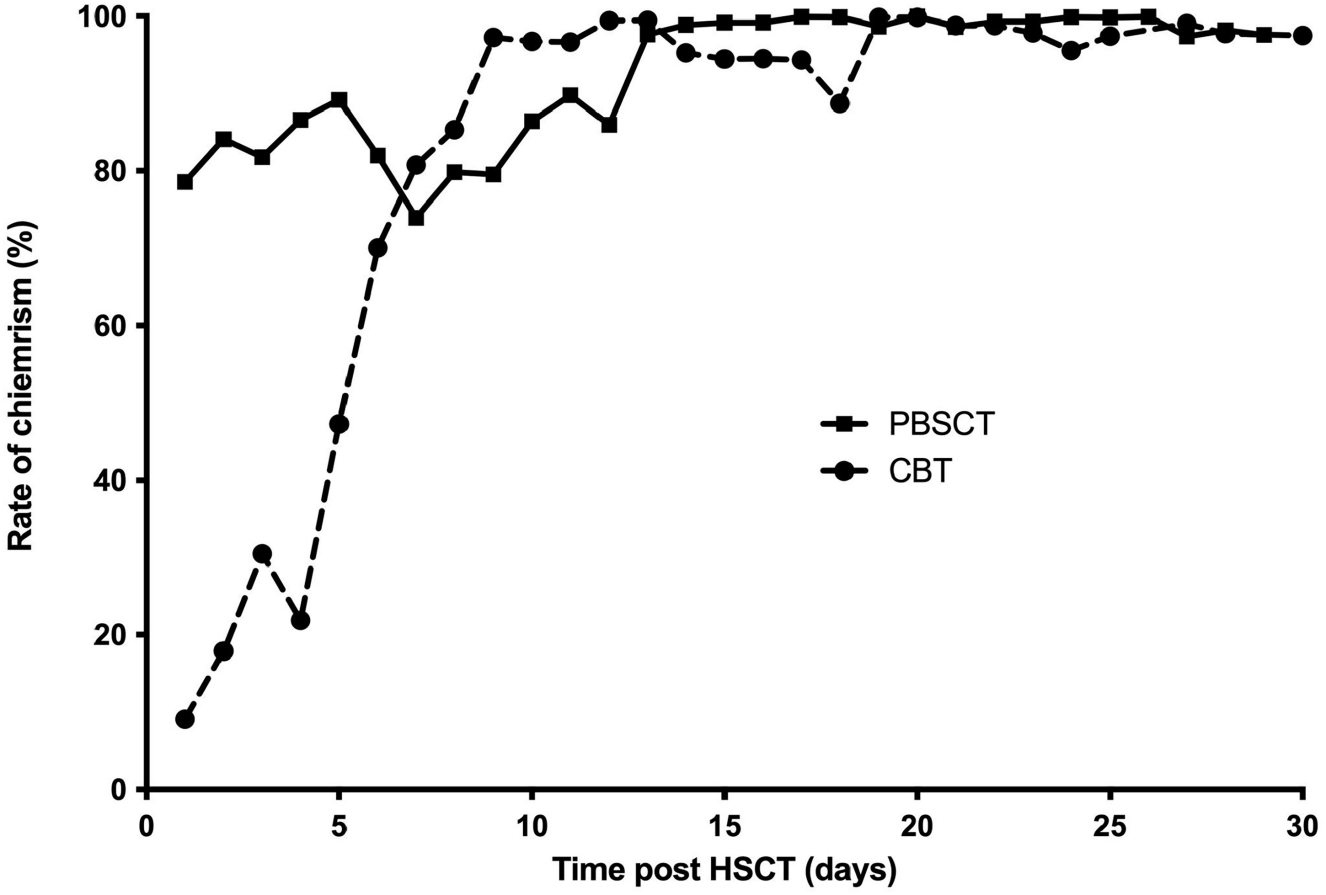
Different patterns of dynamic progress of donor microchimerism in patients received PBSCT and CBT.

Both transplantation types exhibited a clear increase in donor chimerism levels over time. However, early-stage microchimerism trends differed between PBSCT and CBT recipients. In the PBSCT group, chimerism levels rose sharply within the first few days but subsequently fluctuated and gradually declined before stabilizing near 100% donor chimerism. In contrast, the CBT group displayed lower initial chimerism levels (<50% in the first 5 days), followed by a steady, gradual increase. Unlike PBSCT recipients, CBT patients exhibited a more moderate trend without abrupt fluctuations (Figure 2). Consistent with these patterns, the PBSCT group initially showed a higher CDC rate (though not statistically significant), which later converged with that of the CBT group. Additionally, 4 patients in each group maintained constant CDC rates.

### Microchimerism dynamics and clinical outcomes

The median time to achieve CDC was shorter in the PBSCT group (13 days; range, 11–17) than in the CBT group (17 days; range, 9–24, *P* = 0.395). Although not statistically significant, platelet engraftment occurred earlier in the PBSCT group (15 days; range, 10–50) compared to the CBT group (40 days; range, 20–80, *P* = 0.009). Similarly, neutrophil engraftment was marginally faster in PBSCT recipients (14.5 days; range, 11–20) than in CBT recipients (17.5 days; range, 11–28, *P* = 0.165).

Among the 10 deaths, six occurred in the PBSCT and four in the CBT group. The 3- and 5-years OS rates were numerically higher in CBT patients than PBSCT patients (60% vs. 25%, *P* = 0.425), though not statistically significant. The median OS did not differ between groups (*P* = 0.299, Figure 1C), consistent with the stable CDC rates observed in both cohorts.

**FIGURE 3:**
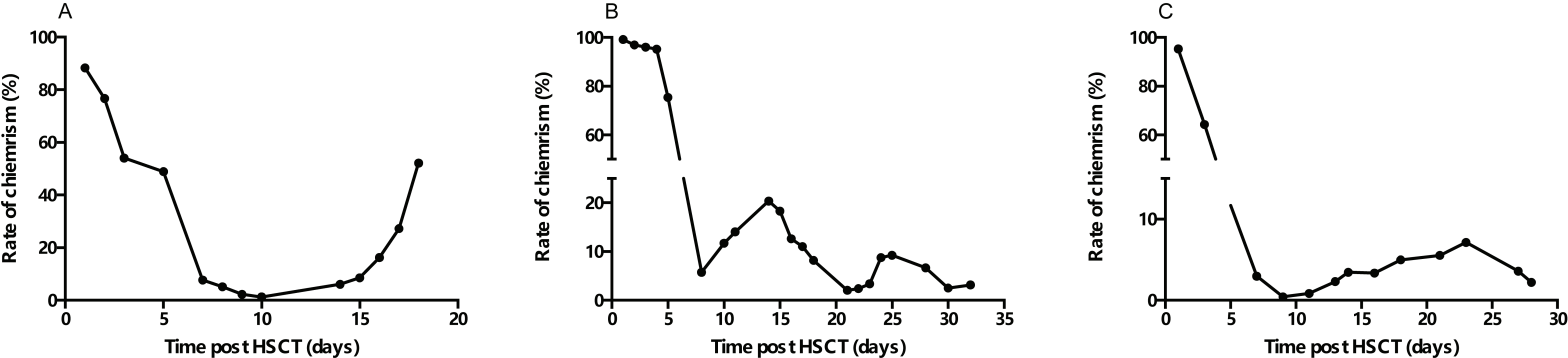
Dynamic progresses of recipient microchimerism levels over time. **(A)** Patient 1; **(B)** patient 8; **(C)** patient 12.

Notably, several cases demonstrated a clear association between microchimerim dynamics and clinical outcomes. For example, patient 1 (AML, CBT) achieved CDC by day +10, followed by neutrophil and platelet engraftment on days +11 and +13, respectively. However, recipient microchimersim rebounded from day +14 onward, leading to relapse by day +30 and death by day +60 despite immunosuppressant withdrawal. Patient 8 (β-thalassemia, CBT) exhibited a decline in recipient microchimerism to 2.03% by day +21, but levels subsequently rose to 8%–9%, and he died by day +56 without achieving CDC. In contrast, patient 12 (WAS, PBSCT) attained CDC by day +9, with neutrophil and platelet engraftment on day +14 and +22, respectively. Despite fluctuating recipient microchimerism (peaking at 7.13% post-day +13) and mixed chimerism, he survived following prompt symptomatic interventions (Figures 3A–C).

## Discussion

The clinical significance of early chimerism assessment as a biomarker for engraftment monitoring remains underexplored in contemporary transplantation literature, despite its potential to refine risk stratification. Our retrospective evaluation of microchimerism dynamics via Indel-qPCR in 18 consecutive allo-HSCT recipients revealed three pivotal findings: (1) an 88.9% CDC rate within 28 days post-transplantation, with 50% achieving sustained CDC, consistent with a prior study (21); (2) a demonstrable correlation between early microchimerism kinetics and hematopoietic recovery; and (3) divergent chimerism trajectories between PBSCT and CBT cohorts, despite equivalent long-term outcomes. Notably, the median time to engraftment demonstrated superiority over historical benchmarks (24–26), a phenomenon attributable to our immunoablative regimen’s efficacy in overcoming host-versus-graft reactions through intensified lymphocyte depletion.

The median 14-days CDC achievement in our cohort, corroborating prior reports (3), establishes a critical temporal benchmark. The observed microchimerism nadir at day 21 with subsequent IMC fluctuations in select cases suggests this timepoint may represent a crucial immunological checkpoint. Furthermore, our data reveal the critical prognostic role of early microchimerism in patients undergoing allo-HSCT. While neutrophil engraftment remained comparable between groups, platelet recovery exhibited a striking association with sustained CDC, with delayed platelet reconstitution significantly more prevalent in patients lacking constant CDC. This divergence extended to long-term survival, where CDC-positive patients demonstrated markedly superior outcomes. These observations align with prior evidence emphasizing regimen-dependent chimerism dynamics in optimizing engraftment and survival (11), while further delineating early microchimerism as a pivotal biomarker for post-transplant risk stratification. Specifically, our data suggest that CDC-negative patients represent a high-risk subgroup warranting intensified monitoring to facilitate preemptive interventions (6). Future studies should explore whether early chimerism-guided therapeutic adjustments, such as timely immunosuppression modulation or donor lymphocyte infusion, could mitigate poor platelet engraftment and mortality in this vulnerable population.

Our study delineates distinct microchimerism kinetics between PBSCT and CBT. PBSCT cohorts exhibited a rapid initial decline in recipient-derived microchimerism, contrasting with the gradual reduction observed in CBT, despite the lower cell counts in cord blood that might predict delayed recovery (27, 28). This divergence correlated with faster hematopoietic reconstitution in PBSCT recipients, suggesting early microchimerism dynamics may serve as a prognostic indicator for short-term engraftment success. Notably, IMC has been implicated in diminished graft-versus-leukemia effects, elevating relapse risk in malignant diseases (28). Thus, real-time chimerism monitoring could guide therapeutic adjustments, such as immunosuppression tapering to augment donor T-cell alloreactivity–a strategy meriting further investigation.

Critically, our results demonstrate that stable engraftment–irrespective of initial cellular dose–can be achieved in the absence of graft rejection, with no discernible disparity in OS between groups. This observation underscores the resilience of hematopoietic recovery under optimized conditioning regimens. The inclusion of cyclophosphamide in our protocol enhanced immunosuppressive potency, particularly in CBT contexts, thereby improving engraftment fidelity. We posit that integrating such regimen refinements with early chimerism surveillance could enable preemptive therapeutic modulation (e.g., donor lymphocyte infusion or immunosuppression adjustment), culminating in superior clinical trajectories.

While this study establishes early microchimerism quantification as a predictive tool for allo-HSCT outcomes, several limitations warrant acknowledgment. The retrospective design, small cohort size, and relatively short follow-up period constrain the generalizability of our findings. Future prospective studies with larger cohorts and extended follow-up diseases are needed to validate the prognostic role of microchimerism dynamics. Additionally, the observed association between early CDC and higher GVHD incidence (7) necessitates further exploration of risk-mitigation strategies, such as tailored immunosuppression protocols.

In summary, this study establishes early microchimerism assessment as a clinically actionable biomarker for engraftment monitoring and risk stratification in allo-HSCT recipients. Our findings demonstrate that: (1) dynamic chimerism patterns, particularly sustained CDC, correlate strongly with hematopoietic recovery and long-term survival; (2) PBSCT and CBT exhibit distinct microchimerism kinetics with prognostic implications for short-term engraftment; and (3) immunoablative regimen optimization, coupled with real-time chimerism surveillance, may enable preemptive interventions to improve outcomes. While the retrospective nature and limited cohort size constrain definitive conclusions, our data compellingly argue for integrating early chimerism monitoring into standard post-transplant care algorithms. Future prospective studies should validate these findings in larger cohorts, explore chimerism-guided therapeutic modulation (e.g., lymphocyte infusion timing), and address the dual challenge of GVHD mitigation while preserving graft-versus-leukemia effects. This work advances the paradigm of precision monitoring in allo-HSCT, where chimerism dynamics may serve as the cornerstone for individualized post-transplant management.

## Data Availability

The original contributions presented in this study are included in this article/supplementary material, further inquiries can be directed to the corresponding author.
